# 2002. Infectious Diseases fellowship training on caring for people who use drugs: A national survey-based needs assessment

**DOI:** 10.1093/ofid/ofad500.129

**Published:** 2023-11-27

**Authors:** Shilpa Vasishta, Raagini Jawa, Sarah Kurz, Nathan Nolan

**Affiliations:** Montefiore Medical Center, New York, NY; University of Pittsburgh, Pittsburgh, Pennsylvania; University of Michigan, Ann Arbor, Michigan; Washington U Sch of Med, St. Louis, Missouri

## Abstract

**Background:**

Infectious complications of substance use are rising nationally among people who use drugs (PWUD), highlighting the need to integrate Infectious Diseases (ID) practice with addiction care. Training experiences among ID fellows, whose practice patterns are likely to be impacted by this syndemic, remain unknown. We conducted a national survey to understand U.S. ID fellows’ training, practices, and perspectives regarding care of PWUD.

**Methods:**

An 18-item survey was developed to assess program characteristics, clinical practices, and perspectives regarding knowledge, comfort, and scope of practice. Recruitment was conducted via emails to fellowship programs, social media (Twitter, Mastodon), and IDSA platforms. Responses were collected over three weeks. Statistical analyses were conducted by chi square test. The study was deemed exempt from institutional review.

**Results:**

196 U.S. ID fellows completed the survey (≅24% response rate), with representation from 9 of 9 geographic regions. All respondents cared for PWUD during their training. 49% had formal curriculum in their programs and 64% reported faculty advocates. 50% typically (in half or more encounters) counseled on harm reduction; 37% referred to outpatient resources and 33% recommended medications for opioid use disorders (MOUD) and naloxone. Respondents were unsure of community resources (e.g. syringe services, 48%; supervised consumption, 57%). 69% rated harm reduction counseling as extremely within scope of ID practice, versus 20% for the practice of recommending MOUD; 25% and 11%, respectively, reported feeling extremely comfortable with these skills. Engagement in discussions of harm reduction, MOUD, naloxone, and outpatient resources was significantly associated with presence of a faculty advocate (p< 0.01 for all).

Self-reported engagement in clinical practices among Infectious Diseases fellows

**Figure:**
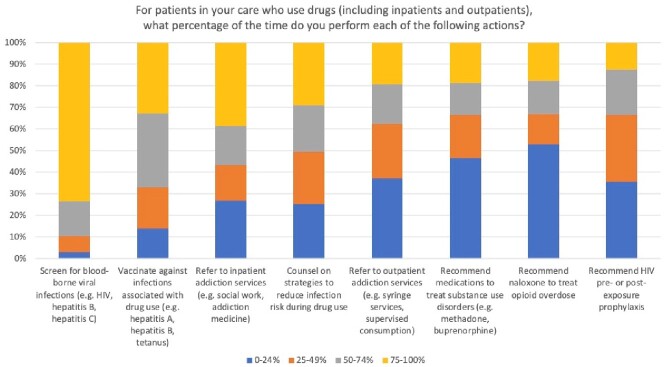

Perspectives on comfort level and scope of practice among Infectious Diseases fellows

**Figure:**
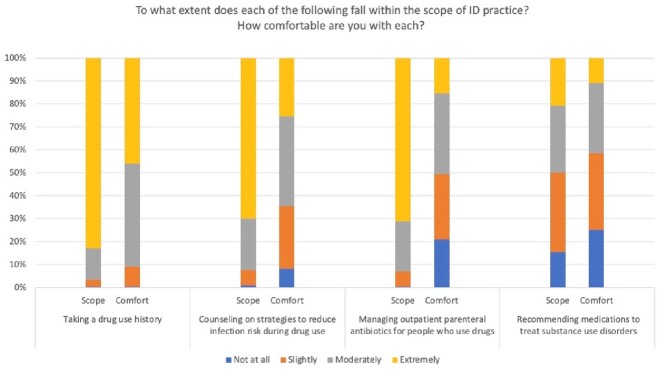

Availability and knowledge of resources at Infectious Diseases training sites

**Figure:**
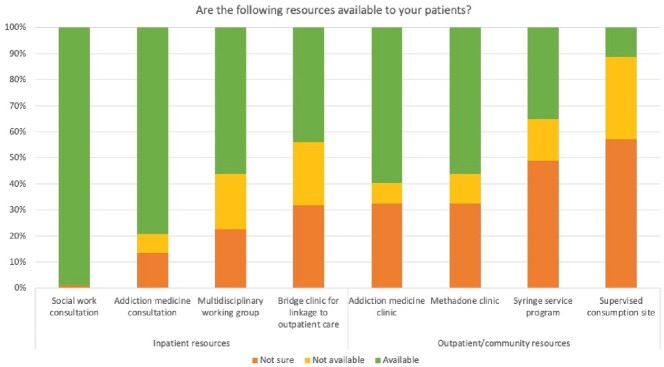

**Conclusion:**

U.S. ID fellows frequently care for PWUD but infrequently engage in integrated care practices. Harm reduction counseling is seen as highly within ID scope of practice, however, comfort and knowledge are low. Both comfort and perception of scope are low for discussing MOUD, though this appears to be impacted by faculty modeling. Our findings highlight an opportunity to formalize training in ID fellowship to better address the need for integrated ID/addiction care nationally.

**Disclosures:**

**All Authors**: No reported disclosures

